# Optimization of *CDT-1* and *XYL1* Expression for Balanced Co-Production of Ethanol and Xylitol from Cellobiose and Xylose by Engineered *Saccharomyces cerevisiae*

**DOI:** 10.1371/journal.pone.0068317

**Published:** 2013-07-02

**Authors:** Jian Zha, Bing-Zhi Li, Ming-Hua Shen, Meng-Long Hu, Hao Song, Ying-Jin Yuan

**Affiliations:** 1 Key Laboratory of Systems Bioengineering (Tianjin University), Ministry of Education, Department of Pharmaceutical Engineering, School of Chemical Engineering & Technology, Tianjin University, Tianjin, P. R. China; 2 School of Chemical and Biomedical Engineering, Nanyang Technological University, Singapore, Singapore; University of Nottingham, United Kingdom

## Abstract

Production of ethanol and xylitol from lignocellulosic hydrolysates is an alternative to the traditional production of ethanol in utilizing biomass. However, the conversion efficiency of xylose to xylitol is restricted by glucose repression, causing a low xylitol titer. To this end, we cloned genes *CDT-1* (encoding a cellodextrin transporter) and *gh1-1* (encoding an intracellular β-glucosidase) from *Neurospora crassa* and *XYL1* (encoding a xylose reductase that converts xylose into xylitol) from *Scheffersomyces stipitis* into *Saccharomyces cerevisiae*, enabling simultaneous production of ethanol and xylitol from a mixture of cellobiose and xylose (main components of lignocellulosic hydrolysates). We further optimized the expression levels of *CDT-1* and *XYL1* by manipulating their promoters and copy-numbers, and constructed an engineered *S. cerevisiae* strain (carrying one copy of *PGK1p-CDT1* and two copies of *TDH3p-XYL1*), which showed an 85.7% increase in xylitol production from the mixture of cellobiose and xylose than that from the mixture of glucose and xylose. Thus, we achieved a balanced co-fermentation of cellobiose (0.165 g/L/h) and xylose (0.162 g/L/h) at similar rates to co-produce ethanol (0.36 g/g) and xylitol (1.00 g/g).

## Introduction

Lignocellulosic materials are renewable, abundant and inexpensive feedstock, which can be used to produce fuels and platform chemicals [1], [2]. Hemicellulose, as a major component of lignocellulosic biomass, can be hydrolyzed to produce xylose, the second most abundant monosaccharide [3]. Xylose together with hexoses (mainly glucose) can be used for ethanol production [4]. Ethanol acts as a substitution of fossil fuels and has many environmental benefits such as minimization of carbon (CO) and nitrogen oxide (NOx) emission. As an alternative, xylose can be used to produce xylitol by microbial fermentation. Xylitol is widely used in medical and food industries, for example as a sugar substitute for diabetic and trauma patients [5], [6], [7]. Moreover, xylitol is a platform chemical that can be used to synthesize many valuable chemicals such as polymers [8]. The price of xylitol (3.4–3.9 USD/kg) in market is much higher than that of ethanol (∼1 USD/kg). Thus, a combined production of ethanol and xylitol is a potential and promising approach to improving the economy of biomass conversion [9], [10], [11].

Xylitol can be produced from xylose through NADPH-dependent xylose reductase (XR) encoded by *XYL1* in yeasts such as *Candida shehatae* [12], [13]. However, the xylitol yield by these yeasts is usually lower than 0.85 g/g xylose due to the fact that xylitol can be further metabolized [6]. Therefore, expression of *XYL1* in *Saccharomyces cerevisiae* can improve the xylitol yield to its theoretically maximum value (1.00 g/g xylose) since xylitol cannot be further used as a carbon source [12], [14].

Xylose utilization in *S. cerevisiae* suffers from glucose repression when glucose and xylose (i.e., main components of lignocellulosic hydrolysates) are both used as feedstock, which is caused by the preferential utilization of glucose [15], [16], [17], [18], [19]. There are no specific transporters for xylose uptake in *S. cerevisiae*. Transport of xylose into cytoplasm is mediated by hexose transporters which show much higher affinities to glucose than to xylose [20]. The discrimination on affinities causes differential uptake and utilization when multiple sugars are present, with glucose having the highest priority, thus leading to “glucose repression”. Such sequential utilization of xylose after glucose exhaustion presents several challenges to xylitol production. Firstly, xylose uptake is tremendously inhibited by glucose. Secondly, insufficient generation of cofactor such as NADPH (after glucose exhaustion) significantly reduces xylitol production since NAD(P)H is required for the conversion. Thus, xylitol production is dramatically impaired by delayed xylose utilization in recombinant *S. cerevisiae*. So far, no effective methods have been proposed to overcome the problems resulting from glucose repression in *S. cerevisiae*.

To increase xylose utilization by recombinant *S. cerevisiae* expressing *XYL1*, repeated fed-batch fermentation was implemented to produce xylitol through continuously supplying glucose under aerobic or anaerobic conditions, but this increased operation costs and could not produce ethanol with high yields simultaneously [21], [22]. A cellobiose metabolic pathway was incorporated into *S. cerevisiae* allowing for the simultaneous co-utilization of cellobiose (a dimer of glucose) and xylose [23], [24]. This approach facilitates faster xylose consumption and ethanol production compared with the traditional method of producing ethanol from mixtures of glucose and xylose, providing a solution to bypassing glucose repression. Furthermore, Jin and coworkers recently cloned the cellobiose metabolic pathway into a recombinant *S. cerevisiae* expressing xylose reductase (*XYL1)* from *Sch. Stipitis* to produce xylitol at a high yield [25]. However, such a strategy of using cellobiose as a co-substrate for xylitol production under oxygen-limited or aerobic conditions may not be optimal because cellobiose can be used as a co-substrate for ethanol production. Xylose uptake decelerates with the decrease of aeration and the uptake rate of xylose is usually lower than that of cellobiose under anaerobic conditions [23], [26], so xylitol production could then be reduced in the anaerobic co-fermentation of xylose and cellobiose. As a result, balanced consumption of cellobiose and xylose is required for efficient co-production of ethanol and xylitol under anaerobic conditions.

**Figure 1:**
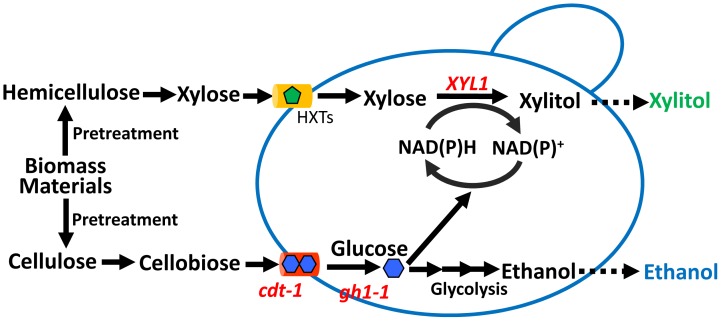
Construction strategy of the recombinant *S.* *cerevisiae* strain to achieve the co-production of ethanol and xylitol from ligonocellulosic biomass. The uptake and hydrolysis of cellobiose (derived from cellulose) were accomplished by a cellodextrin transporter (encoded by *cdt-1*) and an intracellular β-glucosidase (encoded by *gh1-1*), respectively. The uptake and conversion of xylose (derived from hemicellulose) was accomplished by endogenous hexose transporters and a xylose reductase (encoded by *XYL1*), respectively. Thus, ethanol and xylitol were produced simultaneously by this engineered yeast. NAD(P)H for xylitol production was provided by the cellobiose metabolism.

In the present study, we proposed a method to simultaneously produce ethanol and xylitol from mixtures of cellobiose and xylose under anaerobic conditions by construction of a recombinant *S. cerevisiae* strain through expression of a cellodextrin transporter (*CDT-1)* and an intracellular β-glucosidase (*gh1-1)* from *Neurospora crassa* and the xylose reductase (*XYL1)* from *Sch. Stipitis* (Figure 1). In order to balance the consumption of cellobiose and xylose, optimization of the expression of *CDT-1* and *XYL1* was performed by combination of various promoters and copy numbers. The optimization generated an efficient strain SCX-5 that can utilize cellobiose and xylose at similar rates.

## Materials and Methods

### Strains and Media

The strains used in this study are listed in Table 1. The host strain *Saccharomyces cerevisiae* L2612 (*MATα, leu2-3, leu2-112, ura3-52, trp1-298 can1 cyn1 gal+*) was a gift from Professor Thomas Jeffries at University of Wisconsin–Madison. *Escherichia coli* DH5α was used for subcloning and was grown in LB medium supplemented with 100 mg/L ampicillin. Yeasts were grown in SD medium containing 6.7 g/L YNB (yeast nitrogen base), 20 g/L glucose, and 2 g/L amino acid powder mixture (lack of appropriate amino acids whenever necessary). 0.5 mg/L Aureobasidin A (Takara Bio, Kyoto, Japan) was added for the selection of transformants. Anaerobic fermentation was carried out in YPXC medium (10 g/L yeast extract, 20 g/L peptone, 20 g/L xylose, and 20 g/L cellobiose) or YPXG medium (10 g/L yeast extract, 20 g/L peptone, 20 g/L xylose, and 20 g/L glucose). Inoculums were prepared in YPC medium (10 g/L yeast extract, 20 g/L peptone, and 20 g/L cellobiose).

**Table 1:** Strains and plasmids used in the study.

Strains/plasmids	Relevant genotype	Source or reference
**strains**		
L2612	*MAT alpha,leu2,ura3, trp*1	[50]
SCX-1	L2612, *trp1::* pRS304-TDH3XYL1, *ura3::*YIplac211-*cdt-1*, pRS425-ghl-1	This study
SCX-2	L2612, *trp1::*pRS304-PGK1XYL1, *ura3*::YIplac211 cdt-1, pRS425-ghl-1	This study
SCX-3	L2612, *trp1::*pRS304-TDH3XYL1, pRS426-cdt-1, pRS425-ghl-1	This study
SCX-4	L2612, *trp1::*pRS304-PGK1XYL1, pRS426-cdt-1, pRS425-ghl-1	This study
SCX-5	SCX-1, *AUR1*::pAUR101-TDH3XYL1	This study
**Plasmids**		
YIplac211	*URA3*, an integrative plasmid	ATCC 87593
pRS304	*TRP1*, an integrative plasmid	[51]
pAUR101	Aba^r^, an integrative plasmid	Takara
pRS425-gh1-1	Expression of *gh1-1*	[45]
pRS426-cdt-1	Expression of *cdt-1* under *PGK1p*	[45]
pRS304-PGK1	pRS304, *PGK1p*	This study
pRS304-TDH3	pRS304, *TDH3p*	This study
pRS304-PGK1XYL1	Expression of *XYL*1 under *PGK1p*	This study
pRS304-TDH3XYL1	Expression of *XYL*1 under *TDH3p*	This study
pAUR101-TDH3XYL1	Expression of *XYL*1 under *TDH3p*	This study

### Plasmid Construction

The plasmids and primers used in this study are listed in Table 1 and Table S1, respectively. *XYL1* was codon-optimized and chemically synthesized by Geneart AG (Regensurg, Germany). Promoters *PGK1* and *TDH3* were amplified from the genomic DNA of strain L2612 and inserted into pRS304 at *Bam*HI and *Pst*I restriction sites, resulting in the plasmids pRS304-PGK1 and pRS304-TDH3. Subsequently, the synthesized *XYL1* (fused with *PGK1* terminator) was inserted into pRS304-PGK1 and pRS304-TDH3 by *Pst*I and *Xho*I, generating the plasmids pRS304-PGK1XYL1 and pRS304-TDH3XYL1, respectively. The cassette *TDH3p-XYL1* excised from the plasmid pRS304-TDH3XYL1 by *Sac*I and *Xho*I was cloned into plasmid pAUR101 at the sites of *Sac*I and *Sal*I, generating the plasmid pAUR101-TDH3XYL1.

### Strain Construction

The strains harboring a single copy of *CDT-1* were constructed as follows. The *CDT-1* gene fragment with homologous arms to plasmid YIplac211 was amplified using pRS426**-** *cdt-1* as the template (with the primers CDT-F and CDT-R, listed in Table S1). The plasmid YIplac211 was linearized by *Hind*III and *Kpn*I and was then cut into two fragments by *Eco*RV (1000 bp or 2800 bp). The fragments along with the PCR-amplified *CDT-1* fragment were assembled into the *ura3* locus in the chromosome by the DNA assembler method [27], generating the yeast strain with a single copy of *CDT-1*.

The multi-copy plasmid pRS426-*cdt-1* was transformed into yeast L2612 and the strain with multiple copies of *CDT-1* was yielded. Plasmid pRS425-*gh1-1* was transformed, generating the strains capable of utilizing cellobiose (Table 1). The plasmids pRS304-PGK1XYL1, pRS304-TDH3XYL1 and pAUR101-TDH3XYL1 were linearized by appropriate restriction enzymes and transformed, forming strains SCX-1 to SCX-5 as described in Table 1. Yeast transformation was performed by the LiAc/SSDNA/PEG procedure [28].

### Anaerobic Fermentation

Cells were cultivated in YPC medium to prepare inoculums for anaerobic fermentation. Cells at mid-exponential phase were harvested by centrifugation (4,000 rpm, 5 min) and inoculated into 50 mL YPXC or YPXG medium (initial OD_600_ = 1.0) in a 100-mL shaking flask (sealed by a rubber stopper with a syringe needle). Cells were cultivated in a rotary shaker at 30°C and 150 rpm (Honour, Tianjin, China). All fermentations were carried out in duplicates.

### Analysis of Substrates and Fermentation Products

Cell growth was monitored by measuring OD_600_ of cell culture on a Model 722 grating spectrometer (Shanghai No. 3 Analysis Equipment Factory, Shanghai, China). Samples were taken periodically from culture and centrifuged at 10,000 rpm for 5 min. Supernatant was collected for metabolite analysis. Concentrations of sugars, xylitol and ethanol were measured by an HPLC system consisting of an HPLC pump (Waters 1515, Milford, USA), a Bio-Rad HPX-87H column (Bio-Rad, Hercules, CA) and a refractive index detector (Waters 2414, Milford, USA). The column was eluted at 65°C with 5 mM sulfuric acid at 0.6 mL/min.

### Enzyme Assays

To measure XR activity, cells at the mid-exponential phase in the SD medium (with 20 g/L glucose) were harvested and washed twice with ice-cold water. Then, cells were resuspended in the detriethadmine buffer (with 1% PMSF) and disrupted by sonication for 20 min [29]. The protein in the cell lysate was separated by centrifugation for 15 min (4°C, 10,000 rpm). Protein concentration was determined by a Coomassie protein assay kit (Tiangen, Beijing, China). XR activity was measured by the method described previously [30]. The measurement was performed in a solution containing 100 mM Triethanolamine, pH 7.0, 0.2 mM NADPH and 350 mM xylose. Oxidation of NADPH in the reaction was monitored by a spectrophotometer (TU-1900, Persee, Beijing) at 340 nm. One unit (U) of enzyme activity was defined as the amount of NADPH oxidized per minute and the specific activity was defined as units per milligram of protein.

### Data Analysis

A Student’s t-test with two tails was used to calculate the statistical significance of the performances of strains. The statistical function tools of Microsoft Excel was applied to perform the statistical analysis.

## Results and Discussion

### Construction of *S. cerevisiae* Strains Capable of Co-metabolizing Cellobiose and Xylose

To utilize cellobiose, the genes encoding a cellodextrin transporter (*CDT-1*) and a β-glucosidase (*gh1-1*) were introduced into *S. cerevisiae*. Gene *XYL1* encoding xylose reductase was also introduced to convert xylose into xylitol. Cellobiose can be transported into cells via the cellodextrin transporter, hydrolyzed to glucose by the β-glucosidase and used to produce ethanol (Figure 1). Meanwhile, intracellular glucose metabolism can support the conversion of xylose to xylitol by supplying cofactors such as NADPH (Figure 1).

The cellodextrin transporter and xylose reductase are responsible for cellobiose transportation and xylose conversion, respectively. In this study, the gene *gh1-1* was expressed by a multicopy plasmid to guarantee a sufficient activity of β-glucosidase activity, which can avoid accumulation of intracellular cellobiose and metabolic imbalance. Then β-glucosidase is not a main limiting factor in cellobiose metabolism. Thus, only the expression levels of *CDT-1* and *XYL1* were changed to test the impact on productivity and yield of products. Here, we constructed five recombinant *S. cerevisiae* strains, namely SCX-1 to SCX-5 (Table 2). One copy (SCX-1, -2, and -5) or multiple copies (SCX-3, and -4) of *CDT-1* were introduced into the recombinant strains to control its expression level. A relatively weaker promoter *PGK1p* (SCX-2, and -4) or a stronger promoter *TDH3p* (one of the strongest constitutive promoters in yeast [31], in SCX-1, -3, and -5) were incorporated into the recombinant yeasts to control the expression level of *XYL1*.

**Table 2:** Detailed information on the promoter and copy number of the genes *CDT-1* and *XYL1* in the five recombinant strains.

Strain	Promoter/Copy number (*CDT-1)*	Promoter/Copy number (*XYL1)*	Specific XR activity/U/(mg protein)
SCX-1	*PGK1*/1	*TDH3*/1	0.38±0.02
SCX-2	*PGK1*/1	*PGK1*/1	0.16±0.02
SCX-3	*PGK1*/M	*TDH3*/1	0.37±0.07
SCX-4	*PGK1*/M	*PGK1*/1	0.26±0.06
SCX-5	*PGK1*/1	*TDH3*/2	0.73±0.02

M: multiple copies.

The xylose reductase (XR) activities of the five recombinant strains were measured (Table 2). As expected, XR activities in the strains with *XYL1* under the *TDH3p* promoter were higher than those under *PGK1p* promoter. For example, XR activity in SCX-1 was 2.4-fold of that in SCX-2; XR activity in SCX-3 was 1.4-fold of that in SCX-4. Also, a higher copy number of *XYL1* enabled a higher XR activity. Compared with strain SCX-1 which has only one copy of *XYL1*, the specific XR activity of strain SCX-5 with two copies of *XYL1* almost doubled.

### Optimization of the Co-fermentation of Cellobiose and Xylose

**Figure 2:**
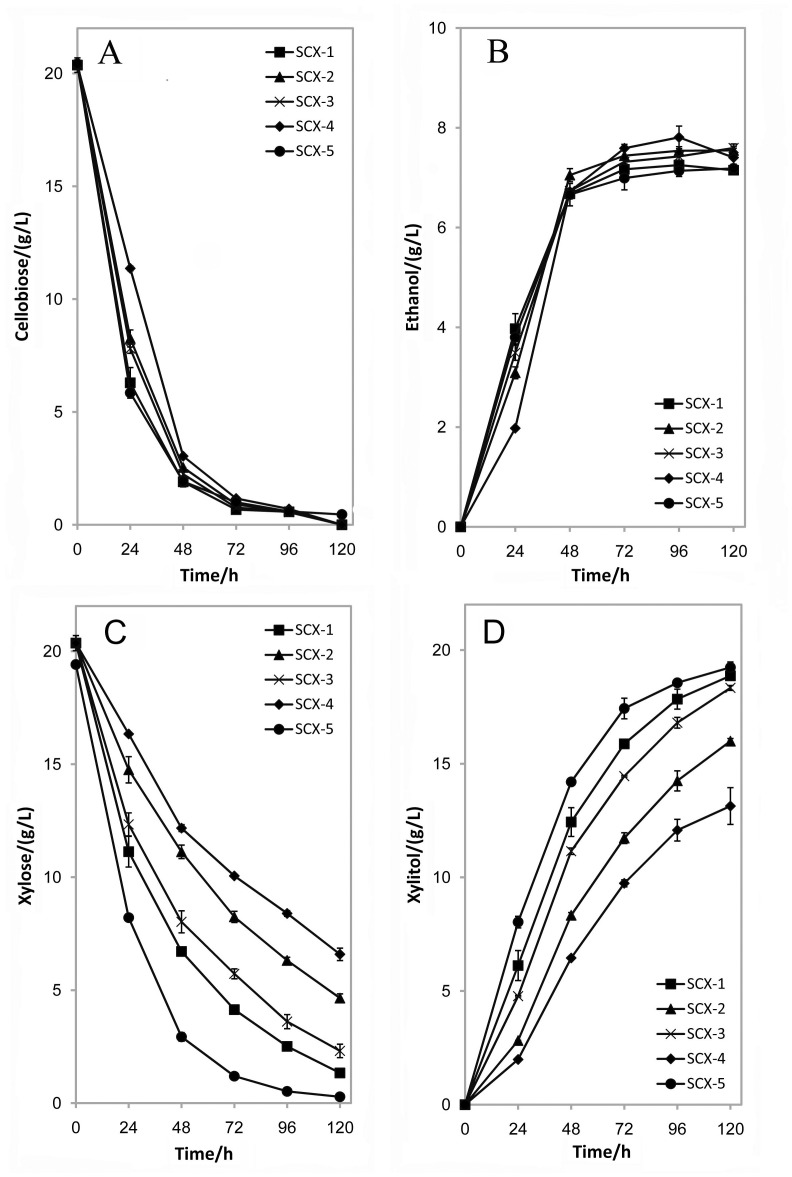
Time profiles of the concentrations of cellobiose (A), ethanol (B), xylose (C) and xylitol (D) by the five recombinant yeast strains (SCX-1 to -5, see **Table 2** **).** The fermentation was conducted under anaerobic conditions in 50 mL YPXC medium containing 20 g/L xylose and 20 g/L cellobiose. The initial cell density was OD_600_ = 1.0. The data was the mean ± standard deviation of duplicate experiments.

To examine the effect of different expression levels of *XYL1* and *CDT-1* on co-fermentation of cellobiose and xylose, we compared the cellobiose and xylose consumption in the five recombinant strains (Figure 2). It was found that the modulation on expression levels of *XYL1* and *CDT-1* had little effect on cellobiose consumption during the whole fermentation process (Figure 2A). Only the initial cellobiose uptake rate during the first 24 h varied a little among the five strains (Figure 2A). We further examined the time profiles of ethanol production by the five recombinant strains, which showed insignificant difference (Figure 2B). Thus, the productivity and yield of ethanol by the five recombinant strains were similar (Table 3).

**Table 3:** Co-fermentation of xylose and cellobiose by the five recombinant strains under anaerobic conditions.

Strains	rxylose(g/L/h)^a^	Ethanol(g/L)^b^	Xylitol(g/L)^b^	Yield(g/g consumed sugars)	
				Ethanol^c^	Xylitol^d^
SCX-1	0.159±0.002	7.15±0.04	18.87±0.12^e^	0.35±0.00	0.99±0.00
SCX-2	0.131±0.002	7.55±0.04	15.99±0.12^f^	0.37±0.00	1.00±0.00
SCX-3	0.150±0.003	7.65±0.08	18.41±0.11^f^	0.37±0.01	1.00±0.00
SCX-4	0.115±0.002	7.40±0.23	13.14±0.81	0.36±0.01	0.95±0.06
SCX-5	0.162±0.000	7.19±0.09	19.24±0.24	0.36±0.01	1.00±0.01

The growth was conducted in 50 mL YPXC medium containing 20 g/L xylose and 20 g/L cellobiose. The initial cell density was OD_600_ = 1.0.

a rxylose: volumetric xylose uptake rate in 120-h fermentation.

b The values were calculated based on the concentrations of metabolites after 120-h fermentation.

c Ethanol yield was represented as g/g consumed cellobiose.

d Xylitol yield was represented as g/g consumed xylose.

e The value was significantly different (*p*<0.05) from SCX-2 and SCX-3.

f The values were significantly different (*p*<0.05) from SCX-4.

In contrast, the expression levels of *CDT-1* and *XYL1* had an evident influence on xylose conversion and xylitol formation. Interestingly, we found that a lower *CDT-1* expression level led to a higher productivity of xylitol (Table 3). For example, SCX-1 and -2 carrying one copy of *CDT-1* had higher xylose utilization rates than those of SCX-3 and -4, respectively. Moreover, a higher expression level of *XYL1* led to a higher xylose utilization and xylitol productivity. SCX-1 and -3 with *XYL1* under the control of *TDH3p* had 21.4% and 30.4% higher xylose consumption rates than SCX-2 and -4 with *XYL1* under *PGK1p*, respectively (Figure 2C, Table 3). Strain SCX-5 derived from SCX-1 bore the highest XR activity. The xylose uptake rate of strain SCX-5 was the highest among the strains (Figure 2C). After 72 h, it consumed 95.1% and 93.8% of the total cellobiose and xylose, respectively. In contrast, the residual xylose of other strains was much higher than that of SCX-5 (Figure 2C). The xylitol production showed consistent profiling with xylose utilization in the five strains (Figure 2D). Therefore, we obtained an efficient strain SCX-5 through fine-tuning the expression of *CDT-1* and *XYL1*.

Optimization of *CDT-1* expression is a useful method to balance the consumption of cellobiose and xylose. Cellobiose transport has been considered to be a limiting factor in cellobiose utilization. In one previous study, overexpression of *CDT-1* increased cellobiose utilization and ethanol production [23]. Another study of improving cellobiose utilization by introduction of protein engineered cellobiose transporters with increased transport kinetics (V_max_) also independently demonstrates the importance of enhancing cellobiose transport [32]. However, excessive overexpression of *CDT-1* might not be beneficial for cellobiose consumption and xylose utilization. A previous study showed that a higher expression level of *Gxf1* (xylose transporter gene) decreased xylose consumption rate compared with its lower expression [33]. Overexpression of *CDT-1* can lead to imbalance between *CDT-1* and gh1-1, which decreases cellobiose consumption. Du and coworkers observed that a more balanced *CDT-1* and *gh1-1* can increase cellobiose utilization [34]. Because cellobiose metabolism supplies NAD(P)H for xylitol formation, decreased cellobiose metabolism will result in insufficient NAD(P)H supply and subsequently will slow down xylose consumption. On the other hand, overexpression of *CDT-1* might cause the excessive occupation of cellodextrin transporters on cell membrane, restricting the normal location of hexose transporters and therefore leading to inefficient xylose uptake. Thus, optimal expression of *CDT-1* is required for efficient co-metabolism of cellobiose and xylose.

Noticeably, it was observed that uptake rates of cellobiose and xylose decreased dramatically when sugar concentration was low (Figure 2A and 2C), which has been reported in literature [19], [35]. Increasing the affinity of cellobiose transporters or hexose transporters to cellobiose or xylose at low sugar concentrations can be a solution to improving the fermentation. Expression of heterologous xylose transporters *Gxf1*, *Sut1* and *At5g59250* can significantly improve transport kinetics in batch cultivation at 4 g/L xylose concentration [36].

Previous studies have demonstrated that higher expression of *XYL1* enables faster xylose fermentation [12], [37], [38]. Integration of one extra copy of *XYL1* increased the xylose consumption rate by 1.7 fold [37]. Matsushika and coworkers also observed that *XYL1* under the control of the promoter *PGK1* enabled 9% more xylose consumption than that under the promoter *ADH1*, which is considered to be a weaker promoter [38]. The increased xylose consumption rate was most probably due to increased xylitol productivity which accelerated the downstream reactions and thus enhanced the carbon flux. In the present study, xylose reductase encoded by *XYL1* catalyzes the conversion of xylose to xylitol. Enhanced expression of *XYL1* resulted in a higher XR activity that could accelerate xylose conversion. A similar result was previously observed in optimization of xylitol production by a recombinant *S. cerevisiae* in a fed-batch culture [39].

Recently, Jin’s group used a similar strategy for enhanced xylitol production [25]. Different from their work, the aim of the present work is coproduction of ethanol and xylitol with high yields from mixtures of cellobiose and xylose rather than just production of xylitol. Additionally, the strategy applied here is combinatorial optimization of the expression levels of *CDT-1* and *XYL1*, which has been proven to be effective in improving the efficiency of fermentation. However, Jin and coworkers did not optimize these genes but enhanced NADPH supply through overexpression of *ALD6*, *IDP2,* and *SsZWF1.*

Taken together, the co-production of ethanol and xylitol were affected by the combinatorial expression of *XYL1* and *CDT-1*, a fine tuning of which enabled the construction of an efficient strain SCX-5 carrying one copy of *CDT-1* and two copies of *XYL1* under the control of *TDH3* promoter. Such an engineered strain SCX-5 could co-utilize cellobiose and xylose at similar rates (∼0.16 g/L/h) and co-produce ethanol and xylitol with high yields (0.36 g/g and 1.00 g/g, respectively).

### Co-fermentation of the Strain SCX-5 in Glucose and Xylose

**Figure 3:**
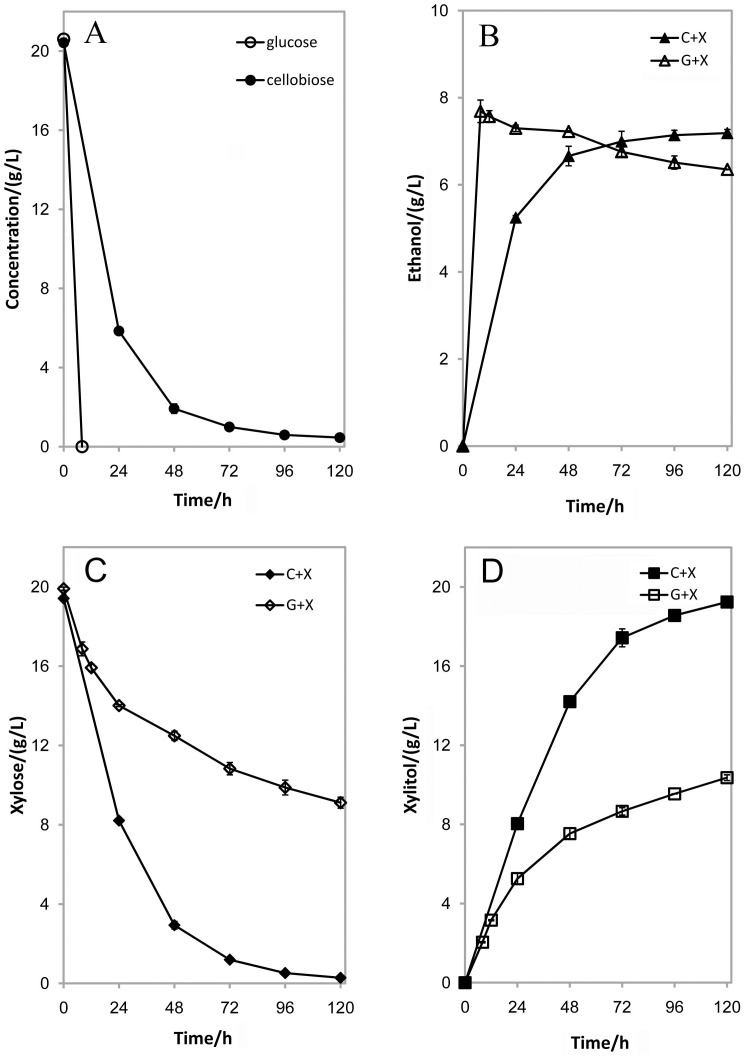
Time profiles of the concentrations of glucose/cellobiose (A), ethanol (B), xylose (C) and xylitol (D) in the co-fermentation of glucose/xylose (G+X) and cellobiose/xylose (C+X) by the engineered strain SCX-5, respectively. The fermentation was performed in 50 mL YPXG medium containing 20 g/L xylose and 20 g/L glucose or 20 g/L cellobiose. The initial cell density was 1.0 (OD_600_). The data was the mean ± standard deviation of duplicate experiments.

To verify that the strategy of co-fermentation by SCX-5 could avoid glucose repression, we investigated the co-fermentation of SCX-5 in glucose and xylose for the co-production of ethanol and xylitol. As shown in Figure 3A, glucose was used up in 8 h. Meanwhile, the ethanol production reached maximum in 8 h with a yield of 0.37 g/g glucose (Figure 3B). The ethanol concentration decreased a little during the following period, which might be due to evaporation or oxidation by SCX-5. However, in the co-fermentation of cellobiose and xylose, the consumption of cellobiose and the production of ethanol were much slower, during which ethanol gradually accumulated in 120 h with a final yield of 0.36 g/g glucose (Figure 3A and 3B).

As for xylose, 98.5% of xylose was consumed in the co-fermentation of cellobiose and xylose (in 120 h), whereas only 54.2% of xylose was used in the co-fermentation of glucose and xylose (Figure 3C). Xylitol production rate was lower in the co-fermentation of glucose and xylose, and the final concentration of xylitol reached only 10.36 g/L (Figure 3D). In contrast, 85.7% more xylitol was produced in the co-fermentation of cellobiose and xylose. The results demonstrated that this strategy of co-utilizing cellobiose and xylose could bypass glucose repression and achieve co-production of ethanol and xylitol at a higher titer.

Diauxic growth pattern is a major obstacle for efficient coproduction of ethanol and xylitol from glucose and xylose in lignocellulosic hydrolysates [14], [40]. To eliminate glucose repression during utilization of multiple sugars in *E. coli*, disruption of *ptsG*, deletion of *cyaA* or introduction of a CRP* mutant (cyclic adenosine monophosphate receptor protein) have been performed [41]. However, these strategies have no effect on relieving glucose repression in *S. cerevisiae*, which has been thought to be related to the competition between glucose and other sugars during uptake process [18]. The entrance of xylose into cells is facilitated by hexose transporters, which show higher preference for glucose than xylose [42], [43], [44]. As a result, xylose uptake is competitively inhibited when glucose is present. In the present study we introduced the cellobiose transporter and β-glucosidase, allowing the utilization of cellobiose so that hexose transporters were solely used for xylose uptake [23], [45]. Therefore, the method applied here provides a way to bypass glucose repression. The efficient ethanol production and xylitol formation proceeded simultaneously from a mixture of cellobiose and xylose, which has not been reported. Although Jin’s group reported a similar method to produce xylitol with high productivity, their design mainly focused on xylitol production rather than ethanol [25]. Our study provides an applicable method to obtain valuable chemicals such as ethanol and xylitol from lignocellulosic hydrolysates, increasing the economic competitiveness of biomass-based biorefinery. In addition, the co-fermentation of cellobiose and xylose in lignocellulosic hydrolysates by SCX-5 can lower the usage of β-glucosidase in cellulase cocktails and thereby reduce the cost associated with the cellulose saccharification [23], [46], [47]. Furthermore, the final products (ethanol and xylitol) can be easily separated by distillation and sequential crystallization, which is a great merit of this strategy [48]. Simultaneous utilization of glucose and xylose was observed for a lipid-producing yeast strain *Trichosporon cutaneum* [49]. However, identification of the specific xylose transporters and successful expression of them in *S. cerevisiae* is difficult to achieve nowadays. Thus, the strategy applied here is still one of the best approaches to overcoming glucose repression.

### Conclusions

In this study, we constructed *S. cerevisiae* strains able to co-produce ethanol and xylitol by expressing the genes encoding cellobiose pathway enzymes (*CDT-1* and *gh1-1*) and the gene encoding XR for xylitol production (*XYL1*). Furthermore, combinatorial modulation of the expression levels of *CDT-1* and *XYL1* resulted in an optimized strain SCX-5 capable of consuming xylose and cellobiose at almost identical rate with the xylitol yield of 1.00 g/g xylose and the ethanol yield of 0.36 g/g cellobiose. SCX-5 produced 85.7% more xylitol in the mixture of cellobiose and xylose than in glucose and xylose.

## Supporting Information

Table S1 **Primers used in the study.**
(DOCX)
