# Supplementary material for: Optimization of CDT-1 and XYL1 Expression for Balanced Co-Production of Ethanol and Xylitol from Cellobiose and Xylose by Engineered Saccharomyces cerevisiae
Source: PLoS One. 2013 Jul 2;8(7):e68317. doi: 10.1371/journal.pone.0068317 (PMC3699558; doi:10.1371/journal.pone.0068317)
Supplement: Table S1 — Primers used in the study. (DOCX) [file pone.0068317.s001.docx]

**Supporting Information**

**Table S1 Primers used in the study.**

| **Primer** | **Sequences** |
| --- | --- |
|  |  |
| CDT-F | TAACAATTTCACACAGGAAACAGCTATGACCATGATTACGCCGTGAGTAAGGAAAGAGTGAGGAA |
| CDT-R | ccagtcacgacgttgtaaaacgacggccagtgaattcgagctcggtaccggccgcaaa |
| CDT-check-F | CATGTCGTCTCACGGCTCCCAT |
| CDT-check-R | TTTACGTCGCCGTCCAGCTCGA |
| PGK1-F | cgcGGATCCGATTCCTGACTTCAACTCAAGACG |
| PGK1-R | gggCTGCAGTGTTTTATATTTGTTGTAAA |
| TDH3-F | cgcGGATCCAACACGCTTTTTCAGTTCGAGT |
| TDH3-R | gggCTGCAGTTTGTTTGTTTATGTGTGTTTATTCG |
| M13F | CAGGAAACAGCTATGACC |
| M13R | TGTAAAACGACGGCCAGTG |
| Xyl1F | GCTCCAGGTGGTAGATTTGTC |
| Xyl1R | GAGCAAATTCGATCAATCTAGGT |
